# Advances in the Treatment of Kidney and Upper Urinary Tract Cancers

**DOI:** 10.3390/biomedicines12030536

**Published:** 2024-02-27

**Authors:** Łukasz Zapała, Paweł Rajwa

**Affiliations:** 1 Clinic of General, Oncological and Functional Urology, Medical University of Warsaw, 02-005 Warsaw, Poland; 2 Department of Urology, Medical University of Silesia, 41-800 Zabrze, Poland; 3 Department of Urology, Comprehensive Cancer Center, Medical University of Vienna, 1090 Vienna, Austria

Kidney and upper tract urinary cancers (UTUC) are diseases of increasing population coverage, the treatment of which is undergoing a continuous process of evolution [1,2]. A characteristic feature of the picture comprising the results of renal cell cancer (RCC) treatment is the significant heterogeneity of patients (including different stages of local advancement and different locations of metastases) [3,4]. Importantly, it is estimated that up to 30% of newly diagnosed RCC cases are patients with disseminated disease at the time of diagnosis [4]. Therefore, predicting the response to modern treatment remains the unmet need of modern urologic oncology [5,6]. Recent advancements in systematic treatment, i.e., immunotherapy, shed additional light on adjuvant treatments to be implemented in new clinical scenarios, including localized disease [7,8].

This Special Issue aims to cover novel approaches in renal and urothelial cancers. We collected papers focused on novel approaches to urothelial cancers involving upper urinary tract and renal cancers that undertook original research focused on pre-clinical studies and biomarkers [9,10,11,12], prognostic models [13,14,15], the specific population of metastatic patients [16], and, finally, two narrative reviews [17,18].

Ito et al., in their paper, presented a novel concept for the search for anticancer drugs [9]. The authors had already reported that the treatment of globotriaosylceramide (Gb3)-expressing cells with catfish (Silurus asotus) egg lectin (SAL) increased the intracellular uptake of propidium iodide (PI) and sunitinib (SU). In their current paper, they tended to determine if SAL pretreatment affects the intracellular uptake and cytotoxic effects of molecular-targeted drugs in RCC cells using commercially available TOS1, TOS3, TOS3LN, and ACHN human RCC cell lines. They revealed that TOS1 cell viability decreased to 70% after treatment with 25 μM SU [18] alone and to 48% after pre-treatment with SAL (50 μg/mL), while there was a significantly enhanced SU uptake noted in SAL-treated TOS1 cells compared to control cells. Interestingly, SAL treatment did not increase PI uptake in normal renal cells. The authors concluded that adequate cytotoxic activity may be achieved even when SU is administered at a sufficiently low dose so as not to cause side effects in combination with SAL.

A recent paper by Szeles et al. [10] focused on the relevance of the circulating soluble levels (sPD-L1) of programmed death ligand-1 (PD-L1) in upper tract urothelial carcinoma (UTUC). The authors prospectively collected serum samples from 61 UTUC patients treated with radical nephroureterectomy (RNU), chemotherapy (CTX), or immune checkpoint inhibitor (ICI) therapy. It was reported that increased pre-operative sPD-L1 was associated with a higher tumor grade (*p* = 0.019) and stage (*p* < 0.001) and the presence of metastasis (*p* = 0.002) in the RNU group. Furthermore, high sPD-L1 levels were significantly correlated with poorer survival in both the RNU- and CTX-treated patients. Finally, the authors showed that ICI treatment caused a significant increase in sPD-L1 (*p* < 0.001). As a consequence, an increased pre-operative sPD-L1 level may act as a predictor of higher pathological tumor stage and worse survival in UTUC, while the specific sPD-L1 flare-up observed in UTUC seems to be therapy-specific, and its clinical relevance needs to be fully assessed.

On the other hand, Bialek et al. [11] investigated the response of renal carcinoma subtypes to the immune checkpoint inhibitor nivolumab in vitro using various RCC cell lines. Increasing doses of nivolumab increased the PD-1 levels in the analyzed cells and caused aggressive behavior in pRCC, but this induced contrary results in ccRCC. All cell lines exhibited a complex response to nivolumab treatment, so the authors summed concluded that there is definitely room for further studies to improve ICI-based therapy for RCC subtypes.

In the study by Velickovic et al. [12], the authors focused on the association of basal compartment and superficial markers, including CK5/6, CD44, CK20, and the pathological characteristics of UTUC associated with Balkan endemic nephropathy (BEN). The study enrolled 127 patients with UTUC who had undergone RNU and extended lymphadenectomy (54 tumors from the BEN region and 73 control UTUC). No significant differences between the expressions of the investigated markers in both groups were observed. The parameters with predictive influence on the expression of CD44 in BEN UTUC included growth pattern (*p* = 0.010), necrosis (*p* = 0.019), and differentiation (*p* = 0.001), and for the control UTUC, lymphovascular invasion (*p* = 0.021). Divergent squamous differentiation in BEN tumors (*p* = 0.026) and stage in control tumors (*p* = 0.049) had a predictive influence on the expression of CK5/6. The authors concluded that BEN and control tumors have a similar antigen presentation of the basal compartment and superficial layer, while the phenotypic characteristics of UTUC have a predictive influence on the expression of the basal compartment and superficial markers.

In the paper by Zapala L. et al. [13], the authors performed an analysis of the relevance of comorbidities and selected inflammatory markers on the survival of patients with primary non-metastatic localized cRCC. In a single-center retrospective cohort of patients post-renal surgery, they included the following parameters in the risk calculations, namely, tumor stage, grade, size, selected hematological markers (SIRI—systemic inflammatory response index; SII—systemic immune-inflammation index), and a comorbidities assessment tool (CCI—Charlson comorbidity index), and then further compared the new concept model with the existing prognostic tools. Four different features were included in the predictive models for CSS (grade, size, stage, and SII) and OS (grade, size, CCI, and SIRI), which were characterized by adequate or even superior accuracy when compared with existing prognostic tools. Here, the authors demonstrated the higher accuracy of their model in OS prediction compared to the commonly used Leibovich and GRANT models, and their model also outperformed the GRANT model in CSS prediction. The described scoring systems can be utilized for the stratification of patients into their respective risk groups for follow-up establishment or enrollment into clinical trials after prospective validation in a larger population.

In another study by Marcinek et al. [14], which included biomarkers in the prognostic models, the authors set out to determine any existing associations between systemic inflammatory reaction syndrome (SIRS) markers and post-operative complications occurrence in patients undergoing kidney tumor surgery and to determine if SIRS occurrence is dependent on age, sex, BMI (body mass index), comorbidities, patients’ general condition before surgery, type of surgery, intraoperative blood loss, or intraoperative ischemia time. They found that BMI values, pre-operative general health status measured with the ASA scale, and the amount of intraoperative blood loss in patients undergoing surgery due to a kidney tumor can contribute to SIRS occurrence. On the other hand, the patient’s sex, age, tumor size, type of surgery, operated side, and time of intraoperative ischemia do not affect SIRS occurrence.

Li et al. [15] focused on the identification of biomarkers of ccRCC and developed a risk model to assess patient prognosis based on the data from the Cancer Genome Atlas (TCGA). They developed a nomogram to predict the prognosis of ccRCC. GO, KEGG, and immunity analyses were used to explore differences in biological function. The authors prepared a risk model containing six BM-associated lncRNAs (LINC02154, IGFL2-AS1, NFE4, AC112715.1, AC092535.5, and AC105105.3), which have been proven to have higher diagnostic efficiency compared to clinical characteristics and can be used to forecast patient prognoses. Then, using RCC cell lines and tissue microarrays to verify the expression of lncRNAs in the risk model, they found that knocking down LINC02154 and AC112715.1 could inhibit the invasion ability of renal cancer cells. The authors concluded that the basement membrane-associated lncRNA risk model may accurately assess the prognosis of individuals with ccRCC.

Semenescu et al. [16] presented a unique cohort with neurosurgical treatment of brain metastases from RCC (BM RCC) (*n* = 24 patients; 91.6% with cRCC and 37.5% after previous nephrectomy). Interestingly, only 29.1% of patients developed extracranial lesions, while 83.3% had a single BM RCC. Neurosurgical resection of the BM was carried out in 23 out of 24 patients. Prognostic factors included previous nephrectomy, having undergone systemic therapy, and a single BM RCC. The authors pinpointed that neurosurgery remains a cornerstone in the treatment of symptomatic BM RCC, being the solution for the quick reversal of neurological manifestations, which, in most cases, can be life-saving.

The recent review by Matuszczak et al. [17] summarized the current treatment options in the management of small renal masses. Briefly, despite the advances in focal methods that have been made in recent years, the NCCN guidelines recommend PN as the preferred method in patients with stage T1a tumors. AS is still recommended in sicker patients, especially the elderly, where surgery is high risk. The authors hypothesized that more long-term and larger cohort-based studies will help to confirm the clinical utility of these methods, together with minimally invasive ablative procedures, and to demonstrate their advantages over classical surgery.

Finally, links between kidney transplant patients and other tumors, e.g., prostate cancer, were presented in the narrative review by Hanusz et al., the first example of the planned thorough analyses on the immunosuppression of cancer patients in these Special Issues [18]. The association between PCa and transplantation is not entirely clear. The authors critically presented a perspective on a possible link between a more frequent occurrence of PCa and a worse prognosis in advanced or metastatic PCa.

In summary, we are witness to constant advances in the modalities used in the management of renal and urothelial cancers [2]. As emphasized in this editorial, the challenges posed by these diseases necessitate a multidimensional approach, including the investigation and integration of novel therapeutic options [4,19]. In the face of these facts, ongoing studies will hopefully lead to improved survival rates, better quality of life, and renewed hope for those affected by renal and urothelial cancers [20].

## References

[1] Trapani D., Curigliano G., Alexandru E., Sternberg C.N. (2020). The global landscape of drug development for kidney cancer. Cancer Treat. Rev..

[2] Chang S.L., Blute M.L. (2023). The Progession Landscape of Diagnostic and Treatment Options for Kidney Cancer. Urol. Clin. N. Am..

[3] Iheanacho K., Vaishampayan U. (2020). Perioperative approaches to kidney cancer. Clin. Adv. Hematol. Oncol..

[4] Graham J., Heng D.Y.C., Brugarolas J., Vaishampayan U. (2018). Personalized Management of Advanced Kidney Cancer. Am. Soc. Clin. Oncol. Educ. Book.

[5] Stewart G.D., Klatte T., Cosmai L., Bex A., Lamb B.W., Moch H., Sala E., Siva S., Porta C., Gallieni M. (2022). The multispeciality approach to the management of localised kidney cancer. Lancet.

[6] Lv Y., Lin S.Y., Hu F.F., Ye Z., Zhang Q., Wang Y., Guo A.Y. (2020). Landscape of cancer diagnostic biomarkers from specifically expressed genes. Brief Bioinform.

[7] Autorino R., Porpiglia F., Dasgupta P., Rassweiler J., Catto J.W., Hampton L.J., Lima E., Mirone V., Derweesh I.H., Debruyne F.M.J. (2017). Precision surgery and genitourinary cancers. Eur. J. Surg. Oncol..

[8] Bedke J., Stuhler V., Stenzl A., Brehmer B. (2018). Immunotherapy for kidney cancer: Status quo and the future. Curr. Opin. Urol..

[9] Ito J., Sugawara S., Tatsuta T., Hosono M., Sato M. (2023). Catfish Egg Lectin Enhances the Cytotoxicity of Sunitinib on Gb3-Expressing Renal Cancer Cells. Biomedicines.

[10] Szeles A., Kovacs P.T., Csizmarik A., Varadi M., Riesz P., Fazekas T., Vancsa S., Hegyi P., Olah C., Tschirdewahn S. (2022). High Pretreatment Serum PD-L1 Levels Are Associated with Muscle Invasion and Shorter Survival in Upper Tract Urothelial Carcinoma. Biomedicines.

[11] Bialek J., Yankulov S., Kawan F., Fornara P., Theil G. (2022). Role of Nivolumab in the Modulation of PD-1 and PD-L1 Expression in Papillary and Clear Cell Renal Carcinoma (RCC). Biomedicines.

[12] Velickovic L.J., Petrovic A.R., Dolicanin Z., Stojnev S., Velickovic F., Basic D. (2024). Expression of Basal Compartment and Superficial Markers in Upper Tract Urothelial Carcinoma Associated with Balkan Endemic Nephropathy, a Worldwide Disease. Biomedicines.

[13] Zapala L., Slusarczyk A., Wolanski R., Kurzyna P., Garbas K., Zapala P., Radziszewski P. (2022). The Four-Feature Prognostic Models for Cancer-Specific and Overall Survival after Surgery for Localized Clear Cell Renal Cancer: Is There a Place for Inflammatory Markers?. Biomedicines.

[14] Marcinek M., Tkocz M., Marczewski K., Partyka R., Kukulski L., Mlynarek-Sniezek K., Sedziak-Marcinek B., Rajwa P., Berezowski A., Kokocinska D. (2023). Evaluation of Parameters Affecting the Occurrence of Systemic Inflammatory Response Syndrome in Patients Operated on Due to Kidney Tumors. Biomedicines.

[15] Li X., Kuang Q., Peng M., Yang K., Luo P. (2023). Basement Membrane-Associated lncRNA Risk Model Predicts Prognosis and Guides Clinical Treatment in Clear Cell Renal Cell Carcinoma. Biomedicines.

[16] Semenescu L.E., Tataranu L.G., Dricu A., Ciubotaru G.V., Radoi M.P., Rodriguez S.M.B., Kamel A. (2023). A Neurosurgical Perspective on Brain Metastases from Renal Cell Carcinoma: Multi-Institutional, Retrospective Analysis. Biomedicines.

[17] Matuszczak M., Kiljanczyk A., Salagierski M. (2022). The Role of Focal Therapy and Active Surveillance for Small Renal Mass Therapy. Biomedicines.

[18] Hanusz K., Domanski P., Strojec K., Zapala P., Zapala L., Radziszewski P. (2023). Prostate Cancer in Transplant Receivers—A Narrative Review on Oncological Outcomes. Biomedicines.

[19] Braun D.A., Bakouny Z., Hirsch L., Flippot R., Van Allen E.M., Wu C.J., Choueiri T.K. (2021). Beyond conventional immune-checkpoint inhibition—Novel immunotherapies for renal cell carcinoma. Nat. Rev. Clin. Oncol..

[20] Hammers H. (2016). Immunotherapy in kidney cancer: The past, present, and future. Curr. Opin. Urol..

